# Genes and Weightlifting Performance

**DOI:** 10.3390/genes13010025

**Published:** 2021-12-23

**Authors:** Naoki Kikuchi, Ethan Moreland, Hiroki Homma, Ekaterina A. Semenova, Mika Saito, Andrey K. Larin, Naoyuki Kobatake, Rinat A. Yusupov, Takanobu Okamoto, Koichi Nakazato, Alun G. Williams, Edward V. Generozov, Ildus I. Ahmetov

**Affiliations:** 1 Graduate School of Health and Sport Science, Nippon Sport Science University, Tokyo 158-8508, Japan; n.kikuchi@nittai.ac.jp (N.K.); hiroki0145@gmail.com (H.H.); sa123ka.v@gmail.com (M.S.); tokamoto@nittai.ac.jp (T.O.); nakazato@nittai.ac.jp (K.N.); 2 Faculty of Sport Science, Nippon Sport Science University, Tokyo 158-8508, Japan; kobatake@nittai.ac.jp; 3 Research Institute for Sport and Exercise Sciences, Liverpool John Moores University, Liverpool L3 5AF, UK; emoreland99@gmail.com; 4 Department of Molecular Biology and Genetics, Federal Research and Clinical Center of Physical-Chemical Medicine of Federal Medical Biological Agency, 119435 Moscow, Russia; alecsekaterina@gmail.com (E.A.S.); zelaz@yandex.ru (A.K.L.); generozov@gmail.com (E.V.G.); 5 Research Institute of Physical Culture and Sport, Volga Region State University of Physical Culture, Sport and Tourism, 420010 Kazan, Russia; 6 Department of Physical Culture and Sport, Kazan National Research Technical University Named after A.N. Tupolev-KAI, 420111 Kazan, Russia; rinatbox@rambler.ru; 7 Faculty of Medical Science, Nippon Sport Science University, Tokyo 158-8508, Japan; 8 Sports Genomics Laboratory, Department of Sport and Exercise Sciences, Manchester Metropolitan University, Manchester M15 6BH, UK; a.g.williams@mmu.ac.uk; 9 Institute of Sport, Exercise and Health, University College London, London W1T 7HA, UK; 10 Department of Physical Education, Plekhanov Russian University of Economics, 115093 Moscow, Russia; 11 Laboratory of Molecular Genetics, Kazan State Medical University, 420012 Kazan, Russia

**Keywords:** strength, weightlifter, athletes, polymorphism, genotype

## Abstract

A recent case-control study identified 28 DNA polymorphisms associated with strength athlete status. However, studies of genotype-phenotype design are required to support those findings. The aim of the present study was to investigate both individually and in combination the association of 28 genetic markers with weightlifting performance in Russian athletes and to replicate the most significant findings in an independent cohort of Japanese athletes. Genomic DNA was collected from 53 elite Russian (31 men and 22 women, 23.3 ± 4.1 years) and 100 sub-elite Japanese (53 men and 47 women, 21.4 ± 4.2 years) weightlifters, and then genotyped using PCR or micro-array analysis. Out of 28 DNA polymorphisms, *LRPPRC* rs10186876 A, *MMS22L* rs9320823 T, *MTHFR* rs1801131 C, and *PHACTR1* rs6905419 C alleles positively correlated (*p* < 0.05) with weightlifting performance (i.e., total lifts in snatch and clean and jerk in official competitions adjusted for sex and body mass) in Russian athletes. Next, using a polygenic approach, we found that carriers of a high (6–8) number of strength-related alleles had better competition results than carriers of a low (0–5) number of strength-related alleles (264.2 (14.7) vs. 239.1 (21.9) points; *p* = 0.009). These findings were replicated in the study of Japanese athletes. More specifically, Japanese carriers of a high number of strength-related alleles were stronger than carriers of a low number of strength-related alleles (212.9 (22.6) vs. 199.1 (17.2) points; *p* = 0.0016). In conclusion, we identified four common gene polymorphisms individually or in combination associated with weightlifting performance in athletes from East European and East Asian geographic ancestries.

## 1. Introduction

Strength is a key factor in success for weightlifters, with success thus highly dependent on the power and force-generating capacity of the muscle [1]. Weightlifting is made up of two lifts, namely a ‘snatch’ and ‘clean and jerk’, with the goal of getting the heaviest “total” from the two lifts. The key contributing factors to performance of strength athletes are skeletal muscle hypertrophy, hyperplasia, fast-twitch muscle fiber predominance, improved neurological adaptation, high glycolytic capacity, and testosterone levels [2,3,4].

Strength athletes display different transcriptomic, biochemical, anthropometric, physiological, biomechanical, and other characteristics comparative to endurance athletes and non-athletic populations [5,6,7,8]. However, little is known about the molecular mechanisms underlying such differences.

Muscle strength/power phenotypes are accepted to be polygenic in nature, in that multiple genetic factors influence the observed phenotype [9,10]. It was suggested that muscle strength and muscle mass have a heritability range of 30–85% [11,12] and 50–95% [13,14], respectively. Overall, these findings provide strong evidence for muscular strength traits to be partly heritable.

Recent reviews suggested that there are at least 38 DNA polymorphisms (single nucleotide polymorphisms; SNPs) associated with strength athlete status [15,16], including those located near or within the *ABHD17C*, *ACTG1*, *ACTN3*, *ADCY3*, *ADPGK*, *AGT*, *ANGPT2*, *ARPP21*, *BCDIN3D*, *CKM*, *CNTFR*, *CRTFR*, *CRTAC1*, *DHODH*, *GALNTL6*, *GBE1*, *GBF1*, *HIF1A*, *IGF1*, *IL6*, *ITPR1*, *KIF1B*, *LRPPRC*, *MLN*, *MMS22L*, *MTHFR*, *NPIPB6*, *PHACTR1*, *PLEKGB1*, *PPARG*, *PPARGC1A*, *R3HDM1*, *RASGRF1*, *RMC1*, *SLC39A8*, *TFAP2D*, *ZKSCAN5*, and *ZNF608* genes. Most of these genetic markers (23 SNPs) were first discovered in the large untrained populations using a genome-wide association study (GWAS) approach [17,18,19] and their association was then replicated in weightlifters and powerlifters, indicating that these markers are the most robust [10]. Overall, studies involving strength athletes and untrained subjects indicate that carriers of gene variants associated with increased muscle mass, an increased proportion of fast-twitch fibers, improved anaerobic metabolism, higher circulating testosterone and neurological adaptation have greater chances of competing at the elite level, due to improved strength potential [15].

Using a case-control study design, we have recently identified 28 DNA polymorphisms (*ABHD17C* rs7165759, *ACTG1* rs6565586, *ADCY3* rs10203386, *ADPGK* rs4776614, *ANGPT2* rs890022, *ARPP21* rs1513475, *BCDIN3D* rs12367809, *CRTAC1* rs563296, *DHODH* rs12599952, *GBE1* rs9877408, *IGF1* rs35767, *IL6* rs1800795, *ITPR1* rs901850, *KIF1B* rs11121542, *LRPPRC* rs10186876, *MMS22L* rs9320823, *MTHFR* rs1801131, *NPIPB6* rs2726036, *PHACTR1* rs6905419, *PLEKHB1* rs7128512, *PPARG* rs1801282, *PPARGC1A* rs8192678, *R3HDM1* rs6759321, *RASGRF1* rs1521624, *RMC1* rs303760, *SLC39A8* rs13135092, *TFAP2D* rs56068671, *ZKSCAN5* rs3843540) associated with strength athlete status [10]. However, studies with a genotype-phenotype design are required to support those findings. The aim of the present study was to investigate individually, and in combination, the association of 28 genetic markers with weightlifting performance in Russian athletes, and to replicate the most significant findings in an independent cohort of Japanese athletes.

## 2. Materials and Methods

### 2.1. Subjects and Ethical Approval

All procedures in this study conformed with ethical standards concerning the scientific research of sport and exercise, and were approved by the local Ethics Committees of the Federal Research and Clinical Center of Physical-Chemical Medicine (protocol #2017/04) and Nippon Sport Science University (protocol #020-G03). Written informed consent was obtained from all participants and the study was conducted in compliance with the Declaration of Helsinki.

Fifty-three elite weightlifters (31 men and 22 women, 23.3 ± 4.1 years) from the Russian cohort (all participants in Olympic Games or World/Europe Championships in 2008–2012) and 100 sub-elite weightlifters (53 men and 47 women, 21.4 ± 4.2 years) from the Japanese cohort (all participants in regional competitions in 2008–2012) participated in the genotype-phenotype study. No subject had ever tested positive for doping. The geographic ancestries of all athletes from Russia and Japan were East European (Caucasians) and East Asian (Japanese), respectively. These cohorts were described in detail in two previous studies [20,21].

### 2.2. Genotyping

Russian samples (*n* = 53) were genotyped using micro-array analysis, while for replication purposes (genotyping of four SNPs) in the Japanese cohort (*n* = 100) we have used TaqMan^®^ SNP Genotyping Assays.

For Russian samples, molecular genetic analysis was performed with DNA samples obtained from leukocytes (venous blood). Four milliliters of venous blood was collected in tubes containing EDTA (Vacuette EDTA tubes; Greiner Bio-One, Kremsmünster, Austria). Blood samples were transported to the laboratory at 4 °C, and DNA was extracted on the same day. DNA extraction and purification were performed using a commercial kit (Techno-Sorb, Moscow, Russia) according to the manufacturer’s instructions (Technoclon, Moscow, Russia). The assay required 200 ng of DNA sample as input with a concentration of at least 50 ng/µL. Exact concentrations of DNA in each sample were measured using a Qubit Fluorometer (Invitrogen, Waltham, MA, USA). The genotyping process was performed using HumanOmni1-Quad BeadChips (Illumina, San Diego, CA, USA) to genotype and impute more than 5 M SNPs, including the 28 SNPs selected for the study (favourable/strength-increasing alleles: *ABHD17C* rs7165759 A, *ACTG1* rs6565586 A, *ADCY3* rs10203386 T, *ADPGK* rs4776614 C, *ANGPT2* rs890022 A, *ARPP21* rs1513475 C, *BCDIN3D* rs12367809 C, *CRTAC1* rs563296 G, *DHODH* rs12599952 A, *GBE1* rs9877408 A, *IGF1* rs35767 A, *IL6* rs1800795 G, *ITPR1* rs901850 T, *KIF1B* rs11121542 G, *LRPPRC* rs10186876 A, *MMS22L* rs9320823 T, *MTHFR* rs1801131 G, *NPIPB6* rs2726036 A, *PHACTR1* rs6905419 C, *PLEKHB1* rs7128512 G, *PPARG* rs1801282 G, *PPARGC1A* rs8192678 A, *R3HDM1* rs6759321 T, *RASGRF1* rs1521624 A, *RMC1* rs303760 C, *SLC39A8* rs13135092 A, *TFAP2D* rs56068671 T, *ZKSCAN5* rs3843540 C). All further procedures were performed according to the instructions of the Infinium High-Density Assay. Ten percent of the samples were genotyped twice with a 100% success rate of reproducibility.

For Japanese samples, the total DNA was extracted and isolated from the saliva of the participants using an Oragene-DNA Kit OG-500 (DNA Genotek, Kanata, ON, Canada). The four polymorphisms, namely *LRPPRC* rs10186876, *MMS22L* rs9320823, *PHACTR1* rs7748390 (in LD with rs6905419) and *MTHFR* rs1801131 were genotyped using TaqMan^®^ SNP Genotyping Assays with the CFX96 TouchTM Real-Time PCR System (Bio-Rad, Hercules, CA, USA). The genotyping mixture (total volume 5 μL) contained 2.5 μL of GTXpress™ Master Mix, 0.125 μL of assay mix (40×), and 1.375 μL of distilled water with 1 μL of genomic DNA (10 ng/μL) per reaction. The thermal cycling conditions included an initial denaturation at 95 °C for 20 s, followed by 40 cycles of denaturation at 95 °C for 3 s and annealing/extension at 60 °C for 20 s. Genotype calls were made based on analysis of the TaqMan^®^ assay results using CFX Maestro Software (Bio-Rad).

### 2.3. Weightlifting Performance Measurement

Evaluation of strength in weightlifters was assessed by their performance in the snatch and clean and jerk (best results in official competitions including Olympic Games, National, Europe, Asian and World Championships). The total mass lifted (in kg) was multiplied by the Wilks Coefficient (*Coeff*) to calculate the standard amount lifted (in point units) normalized across all body mass and sex categories.

$$Coeff=\frac{500}{a+bx+cx^{2}+dx^{3}+ex^{4}+fx^{5}},\;$$

where x is the body mass of the weightlifter in kilograms.

Values for males are: *a* = −216.0475144; *b* = 16.2606339; *c* = −0.002388645; *d* = −0.00113732; *e* = 7.01863 × 10^−6^; *f* = −1.291 × 10^−8^. Values for females are: *a* = 594.31747775582, *b* = −27.23842536447; *c* = 0.82112226871; *d* = −0.00930733913; *e* = 4.731582 × 10^−5^; *f* = −9.054 × 10^−8^.

### 2.4. Statistical Analyses

Statistical analyses were conducted using GraphPad InStat (GraphPad Software, Inc., San Diego, CA, USA) software. Haplotype phasing before imputation was performed using SHAPEIT. Imputation was performed using IMPUTE2. For phasing and imputation, we used 1000 Genomes Phase 3 data as a reference panel and imputed the variants with a frequency higher than 0.1% in the reference panel. Variants imputed with low certainty (info score < 0.6) were filtered out after imputation. Genotype frequencies of athletes were tested for compatibility with Hardy–Weinberg equilibrium (HWE). To evaluate associations between genotypes and weightlifting performance, Spearman rank correlation coefficients were calculated. Differences in strength between different groups (i.e., carriers of high vs. low numbers of strength-related alleles) were analyzed using unpaired *t* tests. Cohen’s D values were calculated as effect estimates for unpaired *t* tests. All data are presented as mean (standard deviation). *p* values < 0.05 were considered statistically significant.

## 3. Results

**Figure 1:**
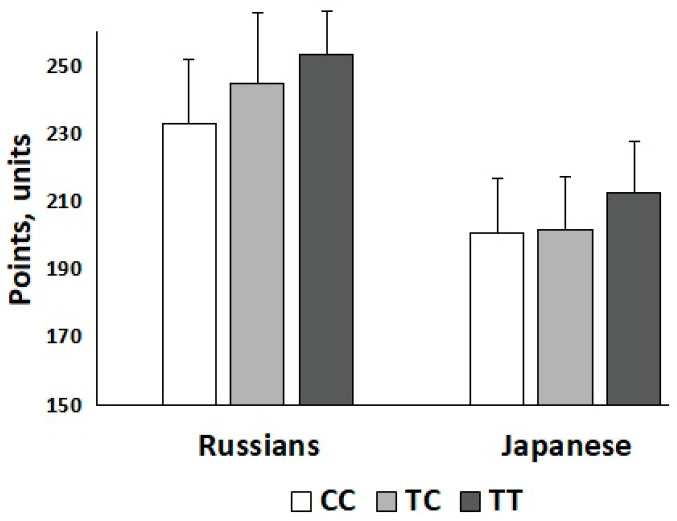
Weightlifting performance in elite Russian and sub-elite Japanese athletes across *MMS22L* rs9320823 genotypes. Statistically significant correlation was found between the number of T alleles and strength in Russian (*r* = 0.33, *p* = 0.017) and Japanese (*r* = 0.20, *p* = 0.047) athletes.

The polygenic score based on all 28 DNA polymorphisms positively correlated (*r* = 0.28, *p* = 0.04) with weightlifting performance (i.e., total lifts in snatch and clean and jerk adjusted for sex and body mass) in Russian athletes. More specifically, we found that carriers of a high (27–34) number of strength-related alleles among Russian weightlifters (58.5%) had better competition results than carriers of a low (20–26) number of strength-related alleles (248.5 (18.0) vs. 232.6 (25.5) points; *p* = 0.01; Cohen’s D = 0.72). Of the 28 DNA polymorphisms, *LRPPRC* rs10186876 A (*r* = 0.30, *p* = 0.026), *MMS22L* rs9320823 T (*r* = 0.33, *p* = 0.017), *MTHFR* rs1801131 C (*r* = 0.27, *p* = 0.048), and *PHACTR1* rs6905419 C (*r* = 0.39, *p* = 0.004) alleles positively correlated with weightlifting performance in Russian athletes (Table 1). Of these four SNPs, only the *MMS22L* rs9320823 T allele was positively correlated with weightlifting performance in Japanese athletes (*r* = 0.20, *p* = 0.047) (Figure 1). The other three SNPs, although not significantly correlated, had the same direction of association in Japanese as in Russian athletes (Table 1).

The distributions of genotypes and allelic frequencies of four SNPs in Russian and Japanese athletes are presented in Table 2. Genotype distributions were in Hardy–Weinberg Equilibrium for both groups of athletes (*p* > 0.05).

Although no association passed Bonferroni correction for multiple testing (i.e., *p* value = 0.05/28 SNPs = 0.0018), we felt justified to use four SNPs in the polygenic analysis given that we used SNPs already discovered independently, most via GWASs at genome-wide significance in untrained subjects and in a case-control design in previous studies. Accordingly, using a polygenic approach, we found that carriers of a high (six to eight) number of strength-related alleles among Russian weightlifters (11.3%) had better competition results than carriers of a low (zero to five) number of strength-related alleles (264.2 (14.7) vs. 239.1 (21.9) points; *p* = 0.009; Cohen’s D = 1.346). These findings were replicated in the study of Japanese athletes. More specifically, Japanese carriers of a high number (28%) of strength-related alleles were stronger than carriers of a low number (72%) of strength-related alleles (212.9 (22.6) vs. 199.1 (17.2) points; *p* = 0.0016; Cohen’s D = 0.687). Given that there were significant differences in allelic frequencies between Russian and Japanese athletes for *LRPPRC* rs10186876 (*p* < 0.0001) and *MTHFR* rs1801131 (*p* = 0.0007), we found that for Russians the best model was to classify athletes using categories of zero to four vs. five to eight alleles. Accordingly, we found that Russian weightlifters with a high number (five to eight) of strength-related alleles (39.6%) were stronger than carriers of a low (zero to four) number of strength-related alleles (258.9 (14.9) vs. 231.6 (20.3) points; *p* < 0.0001; Cohen’s D = 1.533).

## 4. Discussion

In the present study we have demonstrated that four alleles, namely *LRPPRC* rs10186876 A, *MMS22L* rs9320823 T, *MTHFR* rs1801131 C, and *PHACTR1* rs6905419 C, previously identified as markers for strength athlete status (i.e., over-represented in strength athletes vs. controls), were associated with greater weightlifting performance in Russian athletes. The findings for the *MMS22L* rs9320823 T allele were replicated in Japanese athletes. Furthermore, using a panel of those four SNPs, we showed that carriers of a high number of strength-related alleles have better competition results both in Russian and Japanese athletes. Our results expand the existing list of genetic markers associated with weightlifting performance (favourable alleles: *CNTFR* rs41274853 T, *GBF1* rs2273555 A, *MGRN1* rs841194 G, *MLN* rs12055409 G, *ZNF608* rs4626333 G) [4,20,21] and strength performance in bodybuilders (favourable allele: *AR* ≥ 21 CAG repeats) [22].

The *LRPPRC* gene encodes leucine-rich pentatricopeptide repeat cassette protein. It may play a role in cytoskeletal organization, vesicular transport, or in transcriptional regulation of both nuclear and mitochondrial genes by cooperation with *PPARGC1A* [23]. According to the GTEx portal, *LRPPRC* rs10186876 is functional, with the A allele associated with increased expression of the *LRPPRC* gene in skeletal muscle (*p* = 6.2 × 10^−29^). Initially, the *LRPPRC* rs10186876 A allele was reported to be positively associated (*p* = 9.8 × 10^−11^) with handgrip strength adjusted for age, sex, height and BMI in a study of 195,180 individuals [18]. In the follow-up study of Russian athletes, the same allele was over-represented in weightlifters and combined cohort of strength athletes (weightlifters and powerlifters) [10]. The current study now also indicates that the *LRPPRC* rs10186876 AA genotype is associated with greater strength in elite Russian weightlifters.

The *MMS22L* gene encodes methyl methanesulfonate-sensitivity protein 22-like. This protein forms a complex with tonsoku-like, DNA repair protein (TONSL), which recognizes and repairs DNA double-strand breaks at sites of stalled or collapsed replication forks [24]. The encoded protein also can bind with the histone-associated protein NFKBIL2 to help regulate the chromatin state at stalled replication forks [25]. The *MMS22L* rs9320823 T allele was first identified as a marker of increased handgrip strength (*p* = 1.4 × 10^−8^) adjusted for age, sex, and body mass in the UK Biobank cohort (*n* = 334,925) [19]. In the follow-up study of Russian athletes, the same allele was over-represented in highly elite strength athletes (weightlifters and powerlifters) and highly elite weightlifters [10]. In the current study, we now also report that the *MMS22L* rs9320823 TT genotype is associated with greater competition results both in elite Russian and sub-elite Japanese weightlifters.

The *MTHFR* gene encodes methylenetetrahydrofolate reductase protein, one of the key components of folate metabolism. It catalyzes the conversion of 5,10-methylenetetrahydrofolate to 5-methyltetrahydrofolate and may affect global DNA methylation [26]. According to the GTEx portal, *MTHFR* rs1801131 is functional, with the C allele associated with increased expression of the *MTHFR* gene in skeletal muscle (*p* = 5.3 × 10^−12^). In a study of Russian and Polish athletes, the *MTHFR* rs1801131 C allele was over-represented in speed-strength and strength athletes [27]. In the more recent study, the same allele was associated with elite weightlifter status [10]. The current study now also indicates that Russian carriers of the *MTHFR* rs1801131 CC genotype have significantly greater competition results.

The *PHACTR1* gene encodes phosphatase and actin regulator 1 protein. This protein can bind actin and play a role in multiple processes including the regulation of actin cytoskeleton dynamics, actin stress fibers formation, cell motility and survival [28]. The *PHACTR1* rs6905419 C allele was first identified as a marker of increased handgrip strength (*p* = 2.4 × 10^−8^) adjusted for age, sex, and body mass in the UK Biobank cohort (*n* = 334,925) [19]. In the follow-up study of Russian athletes, the *PHACTR1* rs6905419 C allele was over-represented in highly elite strength athletes (weightlifters and powerlifters) [10]. In the current study we now also report that the *PHACTR1* rs6905419 CC genotype is associated with greater strength of elite Russian weightlifters.

We acknowledge the limitations of the present study. First, extension to (we had a limited number of elite and sub-elite weightlifters) and replication within groups of differing geographic ancestry is needed to translate these findings more broadly. Second, none of the associations passed correction for multiple testing, but we felt justified to use four SNPs in the polygenic analysis given that we used SNPs already associated with relevant phenotypes. Of those four SNPs, three (*LRPPRC* rs10186876, *MMS22L* rs9320823, *PHACTR1* rs6905419) were initially found in GWASs, meaning that in the discovery phase (the original articles) these SNPs have passed correction for multiple testing at genome-wide significance (*p* < 5.0 × 10^−8^). The other SNP (*MTHFR* rs1801131) was derived from previous candidate gene studies and associated in at least two previous independent cohorts of athletes. It is common not to adjust for multiple comparisons in the validation phase to prevent the loss of potentially important findings. Third, large studies to evaluate the association of polygenic profile with adaptations in muscle function during training are advocated. In addition, our study is limited to 28 common polymorphisms that were selected from only one study [10]. Accordingly, other SNPs previously identified as markers for strength athlete status (*AGT* rs699, *ACTN3* rs1815739, *CKM* rs8111989, *GALNTL6* rs558129, *HIF1A* rs11549465) [29,30,31,32,33] need to be evaluated for association with weightlifting performance as well. We strongly suspect that many additional common polymorphisms, and probably rare mutations as well, will be shown to be associated with strength performance in due course. Thus, we suspect that the four polymorphisms we have used constitute only a small fraction of the genetic factors that influence human muscle strength. Although these four polymorphisms have been associated with high levels of achievement in weightlifting, we still believe that this is not of sufficient influence to be used in the selection of athletes.

## 5. Conclusions

While many more genetic factors undoubtedly remain undiscovered, our results highlight the relationship between a genetic profile derived from four polymorphisms (*LRPPRC* rs10186876, *MMS22L* rs9320823, *MTHFR* rs1801131, and *PHACTR1* rs6905419) and elite weightlifting performance.

## Figures and Tables

**Table 1:** Weightlifting performance in Russian and Japanese athletes across genotypes.

SNP	Mean (SD)			*r*	*p*
	Genotype 1	Genotype 2	Genotype 3		
*LRPPRC* rs10186876	GG	AG	AA		
Russians	231.8 (21.6)	244.2 (20.6)	251.8 (24.1)	0.30	0.026 *
Japanese	182.5	202.3 (17.3)	203.3 (20.2)	0.03	0.799
*MMS22L* rs9320823	CC	TC	TT		
Russians	233.0 (21.7)	244.8 (23.4)	253.4 (14.2)	0.33	0.017 *
Japanese	200.5 (19.4)	201.4 (19.3)	212.6 (18.9)	0.20	0.047 *
*MTHFR* rs1801131	AA	AC	CC		
Russians	236.1 (21.9)	243.7 (22.8)	254.5 (21.5)	0.27	0.048 *
Japanese	202.4 (18.9)	205.9 (23.3)	202.7 (17.9)	0.05	0.601
*PHACTR1* rs6905419	CC	CT	TT		
Russians	247.1 (22.9)	236.3 (21.5)	224.8 (13.1)	0.39	0.004 *
Japanese	203.2 (21.1)	205.1 (15.9)	195.1 (14.7)	0.08	0.411

* *p* < 0.05, statistically significant correlation between genotypes and weightlifting performance.

**Table 2:** Genotype and allele frequencies of four SNPs in Russian (*n* = 53) and Japanese (*n* = 100) weightlifters.

SNP	Genotype 1	Genotype 2	Genotype 3	Strength-Related Allele Frequency, %
*LRPPRC* rs10186876	GG	AG	AA	A
Russians	17	24	12	45.3
Japanese	1	14	84	91.9
*MMS22L* rs9320823	CC	TC	TT	T
Russians	18	28	7	39.6
Japanese	35	47	17	40.9
*MTHFR* rs1801131	AA	AC	CC	C
Russians	21	26	6	35.8
Japanese	68	25	5	17.9
*PHACTR1* rs6905419	CC	CT	TT	C
Russians	32	17	4	76.4
Japanese	73	23	4	84.5

## Data Availability

The data presented in this study are available on request from the corresponding author.
